# 25, 50 & 75 years ago

**DOI:** 10.1111/ans.17789

**Published:** 2022-06-10

**Authors:** Julian A. Smith

**Affiliations:** ^1^ Department of Surgery Monash University Melbourne Victoria Australia

## Twenty‐five years ago

**Faris IB, Raptis S, Fitridge R. Arterial injury in the lower limb from blunt trauma. *ANZ J. Surg*. 1997; 67: 25–30.**

The present study was performed to identify the factors associated with amputation in patients with blunt injuries to the lower limb associated with arterial injury. The ability of a scoring system to predict the outcome was tested. There were 122 lower limb arterial injuries in 119 patients treated at the Royal Adelaide Hospital in the years 1962–1994. Prognostic factors considered were the site of the injury, the severity of the soft‐tissue injury and shock, the presence of associated injuries and a description of the bone or joint injury. The mangled extremity severity score (MESS) was calculated retrospectively for each patient. The outcome was primary amputation in 27 patients, delayed amputation in 36 patients and limb salvage in 59 patients. The seven deaths were all due to associated injuries. Factors associated with amputation were the severity of shock and soft tissue injury *(P*<0.01), and tibial artery injury compared with more proximal injury *(P*<0.001). Factors that did not affect outcome included delay before repair, method of fracture fixation, or performance of fasciotomy. Amputation was performed in 48/71 (68%) patients with Gustilo type‐IIIC fractures of the tibia. Applying the MESS to our patients resulted in a positive predictive value (PPV) of 71%, a negative predictive value (NPV) of 84% and an overall accuracy of prediction of 75%. The major factor‐determining outcome was the severity of the soft‐tissue injury. Progressive necrosis and infection was a major cause of late amputation. The MESS is not sufficiently precise to allow the decision regarding amputation to be made at the initial operation.

**Turner P, Cocks J, Cade R, Ewing H, Collopy B, Thompson G. Fractured neck of femur (DRG 210/211): prospective outcome study. *ANZ J. Surg*. 1997; 67: 126–30.**

An ageing population will increase the need for resources to treat patients with a fractured neck of femur (DRG 2 I 0/211). Provision of these resources will be helped by a better understanding of current practices. A prospective study of outcome at discharge for 100 consecutive patients with DRG 210/211 was conducted at five Victorian metropolitan teaching hospitals to assess length of stay and the reasons for any variations. The major influences on timing of discharge were delayed availability of rehabilitation beds; the timing of referral and assessment by the Geriatric Assessment Team; delay in surgery more than 24 h after admission; and development of postoperative complications. The efficient management of patients with DRG 2 I 0/211 requires a strong protocol of treatment and referral strategies with adequate resources.

## Fifty years ago

**Conolly WB. Complications following the early treatment of hand injuries: an analysis of 100 cases. *ANZ J. Surg*. 1972; 42: 145–8.**

During 1970 and 1971, 100 major complications of treated hand injuries were referred to the Sydney Hospital Hand Clinic for further treatment. There were 21 cases of necrosis or infection complicating soft‐tissue injuries, 26 cases of severe stiffness complicating fractures and joint injuries, and 39 cases of loss of motor power and sensation complicating tendon and nerve injuries. There were 14 miscellaneous complications. A large proportion of these complications were probably preventable. Common errors and correct treatment are discussed. The application alone of the basic principles as described could probably have prevented a large proportion of the 100 major complications of early treated hand injuries reported in this series. It is the early treatment which decides the final outcome. Skeletal stiffness and failed tendon and nerve repairs may lead to permanent disability. It is far easier to prevent than to cure complications of hand injuries.

**Jairaj PS, O'Brien B McC, Richardson JP, Clarebrough JK, Bennett RC. The experimental application of microsurgical techniques to internal mammary to coronary artery anastomosis. *ANZ J. Surg*. 1972; 41: 379–83.**

Obstructive coronary artery disease is in many patients a disease of the proximal arteries. Saphenous vein bypass grafts have been satisfactorily performed into vessels of diameters of 2.5 mm and above. A certain proportion of patients exist, however, in whom the only vessels available for grafting are of diameters of less than 2 mm. The purpose of this study was to test the feasibility of performing internal mammary to coronary artery anastomosis in the dog in vessels of diameters of 1–2 mm, using the operating microscope. The study indicates that it is technically possible to perform these anastomoses with an acceptable immediate patency rate. The study lends support to the contention of Green that such a technique can be applied clinically and appears to be a logical extension applicable to those cases of coronary artery obstruction where the only distal vessels available for the introduction of a new blood supply are of the order of one to two millimetres in diameter.

## Seventy‐five years ago

**Monk I. An abnormal bronchus arising from the trachea. *ANZ J. Surg*. 1947; 17: 63–5.**

**Fig. 1:**
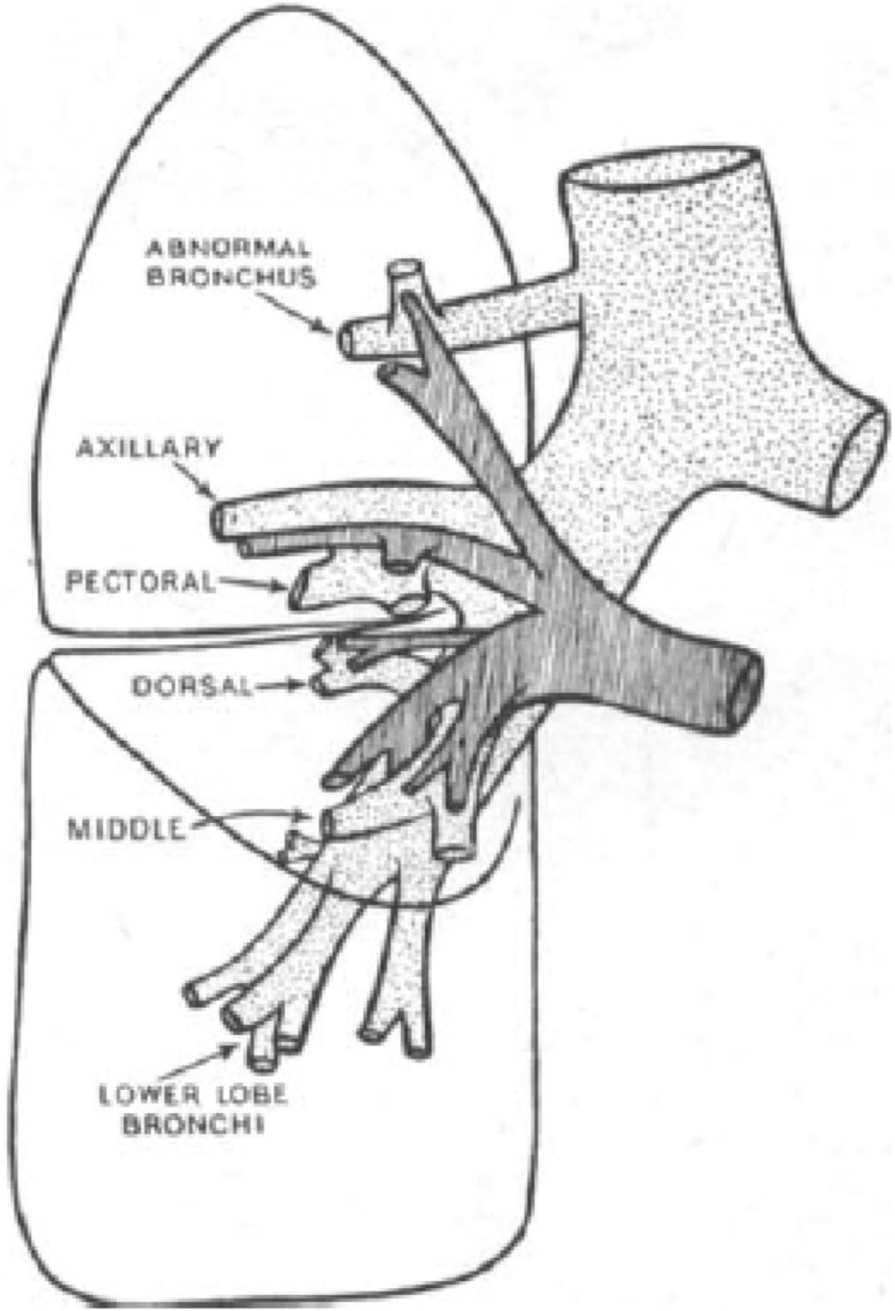
Relation of the abnormal bronchus and pulmonary artery shown.

The steady advance of thoracic surgery and the general acceptance of the dissection technique for removal of lobes and bronchopulmonary segments of the lung, have increased the interest in lung abnormalities. The following abnormality is therefore considered worthy of recording both because of its practical interest and also because of its comparative rarity. The abnormality was accidentally discovered during a dissection of a formalinized lung, the specimen having been obtained from a female, aged twenty‐two years, who died from a subarachnoid haemorrhage. The abnormal bronchus was found arising from the right postero‐lateral aspect of the trachea, 1.5 cm above the bifurcation, and running laterally and very slightly downwards to enter the upper lobe. It was ~1 cm in diameter and 1.5 cm long. The bronchus was distributed to what is normally the apical and subapical bronchopulmonary segments of the upper lobe (Fig. 1). The right upper lobe bronchus arose from the right main bronchus in the normal position, but divided into pectoral and axillary branches only. The right pulmonary artery divided into three main branches, two going to the upper lobe and the third going to the middle and lower lobes; that is, there was an extra ascending branch of the right pulmonary artery which supplied an area of lung corresponding to the apical and subapical segments supplied by the abnormal bronchus (Fig. 1). Not many references were found relative to the described abnormality.

